# Supplementary figures and images for: Phylogenetic relationships and evolutionary history of the greater horseshoe bat, Rhinolophus ferrumequinum, in Northeast Asia
Source: PeerJ. 2016 Oct 11;4:e2472. doi: 10.7717/peerj.2472 (PMC5068396; doi:10.7717/peerj.2472)

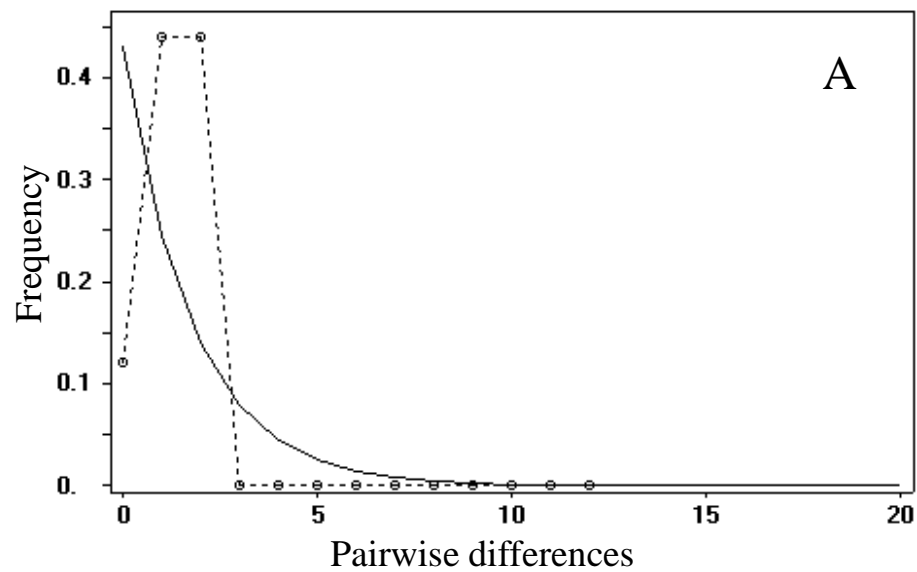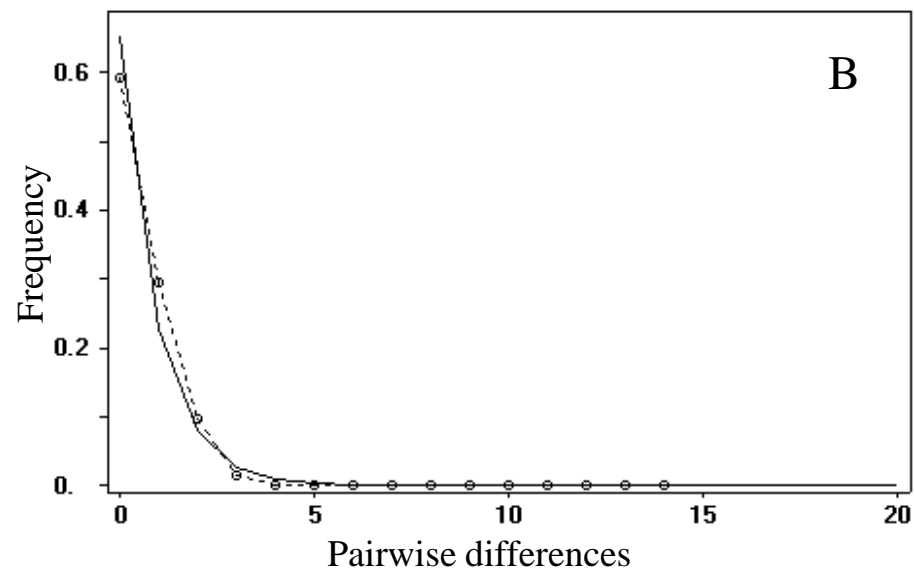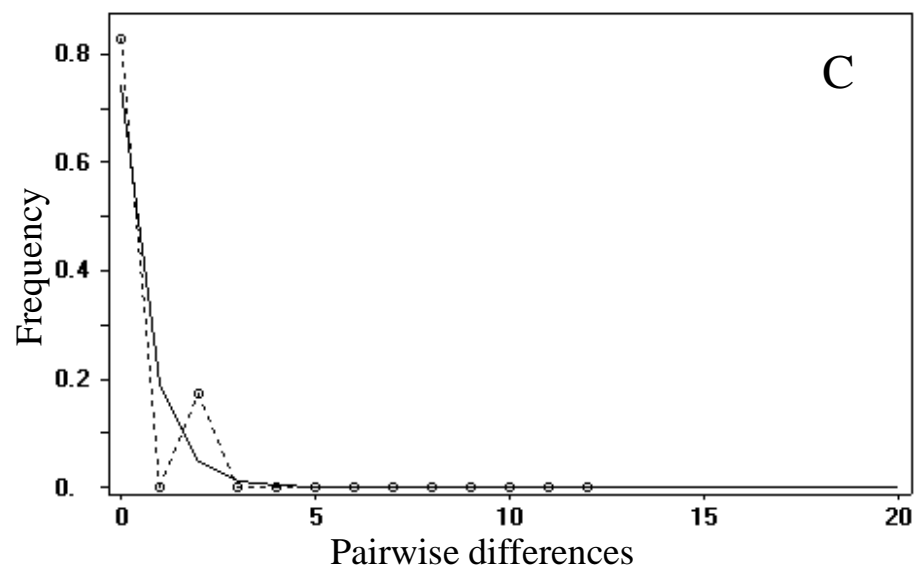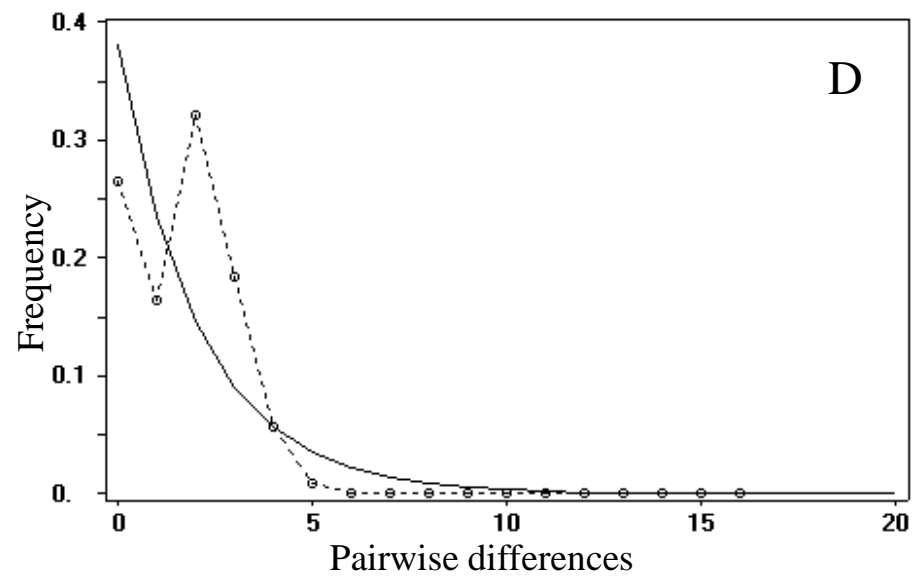

Supplement: Figure S1 — Mismatch distribution of R. ferrumequinum from South Korea (A), Japan (B), Northeast China (C) and Northeast Asia (D). Dashed black lines indicate the observed frequency of pairwise distributions, solid black lines indicate the expected distribution under an expansion model. [file peerj-04-2472-s001.pdf]
