# Supplementary material for: Phylogenetic relationships and evolutionary history of the greater horseshoe bat, Rhinolophus ferrumequinum, in Northeast Asia
Source: PeerJ. 2016 Oct 11;4:e2472. doi: 10.7717/peerj.2472 (PMC5068396; doi:10.7717/peerj.2472)
Supplement: Table S1 [file peerj-04-2472-s002.docx]

**Table S1** Details of sampling localities of the 128 *Rhinolophus ferrumequinum* individuals used in this study.

| **Country/ Province** | **Locality**  **(code)** | **Coordinates** | **Sample number** | | | **Marker** | **GenBank ID (cyt *b*)** | **GenBank ID (D-loop)** |
| --- | --- | --- | --- | --- | --- | --- | --- | --- |
| **Jilin,** | Ji'an (JA) | E125.83° N41.05° | | 14 | cytb+D-loop | | This study | This study |
| **China** |  |  |  | 2 | cytb | | This study | - |
|  |  |  |  | 7 | D-loop | | - | This study |
|  | Liuhe (LH) | E126.00° N42.38° | | 3 | cytb+D-loop | | This study | This study |
|  |  | 5 | D-loop | | - | This study |  |  |
|  | Shuangyang  (ShY) | E125.73° N43.90° | | 4 | D-loop | | - | This study |
| **Liaoning,** | Benxi (BX) | E124.95° N41.38° | | 10 | D-loop | | - | This study |
| **China** |  |  |  | 3 | cytb | | This study | - |
| **South**  **Korea** | Chuncheon (CC) | E127.64° N37.93° | | 1 | D-loop | | - | This study |
|  | Hwacheon (HC) | E127.78° N38.07° | | 5 | D-loop | | - | This study |
|  | Unknown | - | | 1 | cytb+D-loop | | JN392460 | JN392460 |
|  | Unknown | - | | 1 | cytb+D-loop | | NC020326 | NC020326 |
|  | Jeju (JJ) | - | | 6 | cytb | | KP063140 | - |
|  |  |  |  |  |  |  | KP063141 | - |
|  |  |  |  |  |  |  | KP063142 | - |
|  | Naejang (NJ) | - | | 1 | cytb | | KP063143 | - |
|  | Cheongju (CJ) | - | | 3 | cytb | | KP063144 | - |
|  |  |  |  |  |  |  | KP063145 | - |
|  | Yeongweol (YW) | - | | 1 | cytb | | KP063146 | - |
|  | Hwacheon (HC) | - | | 1 | cytb | | KP063146 | - |
| **Japan** | Kashimadai (KM) | - | | 1 | cytb | | AB085721 | - |
|  | Sendai (SE) | - | | 4 | cytb | | AB085721 | - |
|  | Matsuida (MI) | - | | 4 | cytb | | AB085721 | - |
|  | Okutama (OK) | - | | 1 | cytb | | AB085721 | - |
|  | Oshima (OS) | - | | 2 | cytb | | AB085721 | - |
|  | Fujinomiya (FU) | - | | 2 | cytb | | AB085721 | - |
|  | Matsuzaka (MZ) | - | | 5 | cytb | | AB085721 | - |
|  | Shizuoka (SH) | - | | 2 | cytb | | AB085721 | - |
|  | Tenryu (TE) | - | | 3 | cytb | | AB085721 | - |
|  |  |  |  |  |  |  | AB085726 | - |
|  | Kashiwazaki (KW) | - | | 3 | cytb | | AB085721 | - |
|  |  |  |  |  |  |  | AB085729 | - |
|  |  |  |  |  |  |  | AB085731 | - |
|  | Toyama (TO) | - | | 4 | cytb | | AB085721 | - |
|  |  |  |  |  |  |  | AB085727 | - |
|  | Taga (TA) | - | | 5 | cytb | | AB085721 | - |
|  |  |  |  |  |  |  | AB085724 | - |
|  | Tsushima (TS) | - | | 1 | cytb | | AB085721 | - |
|  | Oguchi (OG) | - | | 4 | cytb | | AB085721 | - |
|  | Ohno (OH) | - | | 1 | cytb | | AB085721 | - |
|  | Toyohashi (TY) | - | | 1 | cytb | | AB085728 | - |
| **Other**  **provinces**  **in China** | Henan (HN) | E115.27° N31.87° | | 1 | D-loop | | - | JN230574 |
|  |  |  |  | 4 | cytb | | EF544400 | - |
|  |  |  |  |  |  |  | EF544401 |  |
|  |  |  |  |  |  |  | EF544410 |  |
|  |  |  |  |  |  |  | EF544416 |  |
|  | Shandong (SD) | E117.67° N36.58° | | 1 | D-loop | | - | JN230580 |
|  | Shanxi (SX) | E113.38° N39.33° | | 1 | D-loop | | - | JN230569 |
|  | Shaanxi (SXi) | E106.67° N35.03° | | 2 | D-loop | | - | JN230569 |
|  |  |  |  |  | D-loop | | - | JN230569 |
|  | Beijing (BJ) | E115.72° N39.70° | | 1 | D-loop | | - | JN230562 |
|  | Hubei (HB) | E115.75° N30.67° | | 1 | D-loop | | - | JN230575 |
|  | Guizhou (GZ) | E105.57° N25.28° | | 1 | D-loop | | - | JN230586 |
|  | Yunnan (YN) | E100.72° N22.62° | | 3 | D-loop | | - | This study |
|  |  |  |  |  | D-loop | | - | JN230592 |
|  |  |  |  |  | D-loop | | - | JN230589 |
|  |  |  |  | 2 | cytb | | DQ297575 | - |
|  |  |  |  |  |  |  | EU434936 | - |

­**Lon, longitude; lat, latitude;** -, missing data.
