# Supplementary material for: Phylogenetic relationships and evolutionary history of the greater horseshoe bat, Rhinolophus ferrumequinum, in Northeast Asia
Source: PeerJ. 2016 Oct 11;4:e2472. doi: 10.7717/peerj.2472 (PMC5068396; doi:10.7717/peerj.2472)
Supplement: Table S2 [file peerj-04-2472-s003.docx]

**Table S2** Haplotypes of the greater horseshoe bat, *Rhinolophus ferrumequinum*, in Northeast Asia.

| **Haplotype** | **N** | **Locality** | **Haplotype** | **N** | **Locality** |
| --- | --- | --- | --- | --- | --- |
| **Cyt *b*** |  |  |  |  |  |
| H1  H2  H3 | 33  4  2 | Japan  Japan  Japan | H9 | 1 | Korea |
|  |  |  | H10 | 2 | Korea |
|  |  |  | H11 | 1 | Korea |
| H4 | 1 | Japan | H12 | 2 | Korea |
| H5 | 1 | Japan | H13 | 1 | Korea |
| H6 | 1 | Japan | H14 | 2 | LH |
| H7 | 1 | Japan | H15 | 24 | JA (16)/LH (1)/  BX (3)/Kor (4) |
| H8 | 3 | Korea |  |  |  |
| **D-loop** |  |  |  |  |  |
| H1  H2  H3  H4  H5  H6 | 1  1  1  4  3  1 | Korea  Korea  Korea  JA  JA  JA | H7  H8  H9  H10 | 1  2  4  33 | BX  BX  BX (1)/JA (3)  JA (10)/LH (8)/  SY (4)/BX (6)/  Kor (5) |

Here Kor and Korea refers to South Korea;

N, individual numbers, the same with numbers in the brackets;

locality codes are described in Table S1.
